# Frozen Elephant Trunk: Technical Overview and Our Experience with a Patient-Tailored Approach

**DOI:** 10.3390/jcm11041120

**Published:** 2022-02-20

**Authors:** Alan Gallingani, Andrea Venturini, Matteo Scarpanti, Domenico Mangino, Francesco Formica

**Affiliations:** 1 Cardiac Surgery Unit, University Hospital of Parma, 43126 Parma, Italy; agallingani@ao.pr.it (A.G.); matteo.scarpanti@gmail.com (M.S.); 2 Cardiac Surgery Unit, Angel Hospital, 30174 Mestre, Italy; andrventurini@libero.it (A.V.); domenico.mangino@aulss3.veneto.it (D.M.); 3 Department of Medicine and Surgery, University of Parma, 43126 Parma, Italy

**Keywords:** frozen elephant trunk, aortic arch replacement, aortic dissection, aortic arch hybrid prosthesis, aortic surgery, endovascular arch surgery

## Abstract

Ever since the first hybrid prosthesis was used for a total aortic arch replacement, many other techniques have been developed to comply with the need for the treatment of a wide spectrum of patients and their clinical pictures. We hereby provide an overview of the most popular surgical techniques to perform a frozen elephant trunk, including our tailored approach revolving around the antegrade deployment of a Gore C-TAG endovascular stent graft sutured to a four-branched vascular prosthesis. This technique was applied to three cases of acute type A aortic dissection. Although our small series of patients consists of acute aortic dissections only, this technique could be applied to any other aortic arch pathology, such as chronic dissections or aneurysms. Moreover, we believe that, because of the individually tailored approach and widespread availability of the necessary materials, this technique can reveal itself useful in many different operative scenarios.

## 1. Introduction

The treatment of complex aortic pathologies remains, to date, one of the most significant challenges for cardiovascular surgeons. With the advent of new technologies and new strategies, we have come a long way in approaching the wide spectrum of aortic diseases. The purpose of this article is to provide an overview of the most widely used techniques for total aortic arch replacement as well as providing a variant frozen elephant trunk strategy that we have developed, with the combination of a C-TAG thoracic stent graft (W.L. Gore & Associates, Flagstaff, AZ, USA) used as the frozen trunk and a 4-branch Hemashield Platinum vascular graft (Maquet, Rastatt, Germany). 

Up to the early 2000s, such pathologies involving both the aortic arch and the descending thoracic aorta were mainly treated by a two-stage approach, the elephant trunk technique described in 1983 by Borst [1]. Although this technique evolved over time [2,3], the most significant breakthrough has been the introduction of a hybrid prosthesis consisting of a distal endovascular stent graft and a proximal conventional surgical graft. The modified approach was named the frozen elephant trunk (FET) technique [4]. Due to the more streamlined nature of the procedure, which greatly simplified the treatment of aortic pathologies, the FET procedure gained widespread popularity. The FET is potentially indicated for all pathologies of the aortic arch, aneurysms and dissections. The endoluminal sealing of the surgical suture line by the stent graft improves haemostasis and makes the frozen elephant trunk technique ideal to fix fragile aortic tissues.

Current consensus identifies four main procedures in the setting of extended arch replacement [5]: (1) total arch replacement with or without the standard elephant trunk (ET), without deploying a stented graft in the thoracic descending aorta; (2) total arch replacement and descending thoracic aortic stent grafting with frozen stented graft placed under circulatory arrest; (3) hemiarch replacement and descending thoracic aortic stent grafting with the stent graft placed under circulatory arrest; (4) total arch replacement with stent graft placed after completion of cardiopulmonary bypass and with the use of fluoroscopy to identify the landing zones. Of these four techniques, the following discussion will focus on the procedures that include the deployment of a stented graft in the descending thoracic aorta.

Current guidelines highlight the importance of the FET technique in the treatment of aortic arch pathologies when compared to just ET [6]. Since the inception of the aforementioned procedure, surgeons have devised ways to simplify and address various techniques to perform the FET. 

The original ET technique relied on a two-stage procedure, where the second stage consisted of an open total descending aortic replacement with an excessively high rate of mortality and operation-related morbidity. In addition to this, many patients did not manage to survive to the second stage procedure or the risk associated with it was deemed too high after the first stage.

To comply with the need for a definitive one-stage operation, new generation hybrid prostheses entered the field, combining a total-arch branched graft with an endovascular deployable stent. Of these, there are two devices currently widely used in European countries: the Terumo Aortic Thoraflex Hybrid (Vascutek Terumo, Inchinnan, Scotland, UK) [7] and the E-Vita Open Neo (Jotec Gmbh, Hechingen, Germany) [8]. Other prostheses available on the market are the Cronus (MicroPort, Shanghai, China) and the Frozenix (Japan Lifeline, Tokyo, Japan) [9,10].

As the FET procedure has evolved, there have been many variations on the original technique, while keeping the core idea of a stented graft delivered in an antegrade fashion and fixed into position, preventing the risk of proximal endoleaks. The antegrade deployment of a stented graft provides a safe landing zone for future distal extensions. In most scenarios, FET deployment, at least at the transition zone 3–4, provides a safe length for additional stent grafts and easier retrograde access [6]. 

Has the widespread use of hybrid prostheses rendered any other technique obsolete? The goal of this article is to bring particular attention to settings where immediate availability of hybrid grafts is not possible or where a custom tailor-made approach is the determining factor for the success of a very complex surgical task. 

In countries where hybrid prostheses are not licensed for medical use (i.e., the United States, where, so far, they have not obtained FDA approval) or are not commercially available, the two options for performing an FET procedure are direct antegrade placement of aortic stent grafts through the open aorta under systemic circulatory arrest and a hybrid procedure consisting of an open surgical replacement alongside a totally endovascular graft placement. In the first scenario, to ensure its accurate placement, the proximal edge of the stented graft is sutured in place and secured to the native aorta with or without direct modifications to the graft device [11]. There are leading aortic teams worldwide advocating for a two-staged approach with complete or partial debranching of epiaortic vessels and replacement of the ascending aorta and aortic arch, utilising the vascular prosthesis as a landing zone for a second endovascular completion [12]. 

In this article, we present the essentials of a procedure that we have devised to address the issue of performing a complete frozen elephant trunk total arch replacement in a setting where a hybrid prosthesis is not readily available for use, especially in emergent scenarios. Our technique involves the antegrade implantation of a Gore C-TAG endovascular graft into the distal aortic arch, following the removal of the diseased portion of the aorta (ascending aorta and proximal aortic arch) and prior to the debranching of the epiaortic vessels. The proximal end of the endovascular graft is then secured to the native aorta with a Teflon-reinforced suture line to strengthen the anastomotic site. This suture is usually performed between the innominate and the left carotid artery (zone 1) or between the left carotid artery and the left subclavian artery origin (zone 2) to help with bleeding control, also reducing the risk of laryngeal nerve palsy. Subsequently, to this stump, we suture the distal end of a 4-branched vascular Dacron graft in order to complete the frozen elephant trunk, thereby achieving a total arch repair with a tailor-made hybrid prosthesis composed of two portions that are individually measured and deployed.

## 2. FET Techniques

Current evidence suggests that the frozen elephant trunk procedure should be considered in all cases of extensive aortic arch pathologies and in which the presence of an endovascular stent graft in zone 3–4 allows both definitive treatment and a safe landing zone for future endovascular completion. 

### 2.1. Technique from Asia

In 2013, Ma et al. reported their results using the Sun’s procedure for the frozen elephant trunk [13]. This technique consists of an antegrade deployment of the stent graft under circulatory arrest as a first step. Moreover, the Cronus™ graft consists of a regular Dacron vascular prosthesis and interconnected Z-shaped stents made from conichrome (a Co-Cr-Ni-Mo-Fe alloy). At the proximal and distal ends, there is an extra centimetre of Dacron sewing cuff which can be helpful in performing a standard surgical anastomosis. Because of its technical specifications, the prosthesis can be easily crimpable and quickly deployable. Subsequently, any vascular graft can be attached to the proximal sewing cuff to complete the frozen elephant trunk procedure. 

Similarly, the Frozenix™ prosthesis can be deployed in an antegrade manner and then secured to the native aorta and any vascular prostheses with a direct suture. This graft is a self-expandable nitinol wire enclosed in a malleable introducer sheath; it varies in size and length and before deployment its curvature can be adjusted [14].

These two prostheses are used in few nations worldwide and the majority of these devices have been employed in their countries of manufacturing, China and Japan, respectively.

### 2.2. Hybrid Techniques from the US

In the US, Bavaria et al. describe the FET technique utilising a stent graft delivered on a beating heart into the proximal descending aorta [15]. A complete FET is achieved by performing a type I or type II surgical debranching of the epiaortic vessels (creating an optimised landing zone to anchor the stented graft) followed by a stent graft deployment in the aortic arch and proximal descending aorta in an antegrade fashion utilising a secondary side-branch of the vascular prosthesis or in a retrograde fashion through the femoral artery. 

Another hybrid approach consists of surgical replacement of the ascending aorta and total endovascular arch stenting either with antegrade or retrograde deployment without debranching of the epiaortic vessels [16,17].

### 2.3. Ready-Made FET from the EU

From the first prototype of the Chavan–Haverich prosthesis, the idea of a ready-made hybrid vascular graft gained widespread approval [18]. In later years, based on the same principle, in Europe, the Evita Open (Jotec Gmbh, Hechingen, Germany) and the Thoraflex Hybrid (Vascutek Terumo, Inchinnan, Scotland, UK) were developed. The two devices were launched on the market with several paired diameters of the vascular and stent graft. Both prostheses carry similar technical specifications and the same surgical deployment: the stent graft is collapsed into a delivery system and it is then deployed under systemic circulatory arrest, in direct vision, as the first step of the surgical procedure. Subsequently, the collar part of the prosthesis is then anastomosed onto the native aorta to also provide optimal sealing in instances of dilated aortic arches caused by dissections or aneurysms. According to the clinical scenario and surgeon preferences, different conformations of these devices can be used: single side-branch (Island technique), 4-branched vascular prosthesis or the more recent single trifurcated vascular graft (selective epiaortic vessels reimplant). 

The completion of the FET operation is achieved by performing the proximal anastomosis and the reimplantation of the epiaortic vessels, either maintaining their anatomical origin (Island technique) or by selective reimplant [19]. 

### 2.4. Our Tailored FET Technique

We hereby report our technique utilised in three patients who underwent an FET procedure with an individually tailored approach. For all patients, preoperative CT scans showed an acute type A aortic dissection with the primary entry tear located in the aortic arch. The extension of the dissection for patients 1 and 2 was from the sinotubular junction (STJ) to the iliac vessels while for patient 3 it was from the STJ to the proximal descending aorta. Patients were 55, 54 and 77 years old, respectively. The arterial cannulation site was femoral in order to initiate cooling and to perfuse the lower body while performing the distal anastomosis. In this manner, systemic circulatory arrest time is limited only to the deployment of the endovascular graft. Visceral perfusion is then achieved by inserting a Foley catheter directly into the endoprosthesis and initiating a low-flow (1–2 litres per minute) consistent oxygenation of the lower body. 

After institution of cardiopulmonary bypass (CPB), the aorta was cross-clamped and retrograde cold blood St. Thomas cardioplegia was delivered. During systemic cooling, the proximal ascending aorta was resected to the STJ. Aortic valve replacement with a bioprosthesis was performed in the third patient only. Once 25 °C nasopharyngeal temperature was reached, circulatory arrest and antegrade selective cerebral perfusion (ASCP) by means of True Flow RDB cannulas (Med Europe SRL, Bologna, Italy) placed in the innominate and left carotid arteries were initiated. The aorta was further resected between the innominate and the left common carotid artery. The epiaortic vessels were transected and their ostia were carefully sutured. 

**Figure 1:**
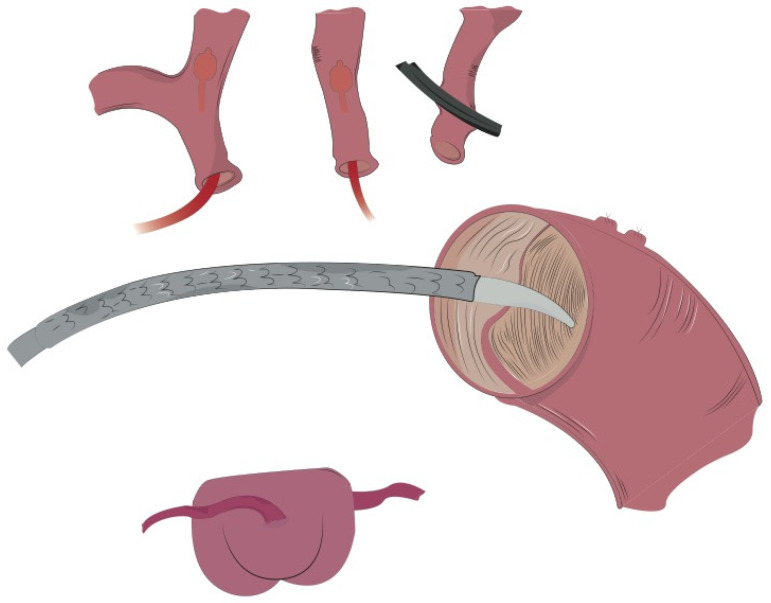
Under moderate hypothermic circulatory arrest, the aortic arch was resected to zone 1 and antegrade selective cerebral perfusion was promptly instituted. Then, the previously selected GORE CTAG was inserted into the true lumen of the aortic arch.

The diameter of the proximal descending aorta was measured previously, from the preoperative CT scan. A 150 mm Gore C-TAG prosthesis was chosen without oversizing. Following the experience of Tsagakis [20], we routinely used angioscopy during the procedure, which provided us with a clear vision of the anatomy of the aorta before deploying the endovascular prosthesis and also allowed us to evaluate the correct positioning and opening of the stent graft; hence, the endoprosthesis was deployed into the true lumen under direct vision without the need for a supporting guide-wire (Figure 1). 

**Figure 2:**
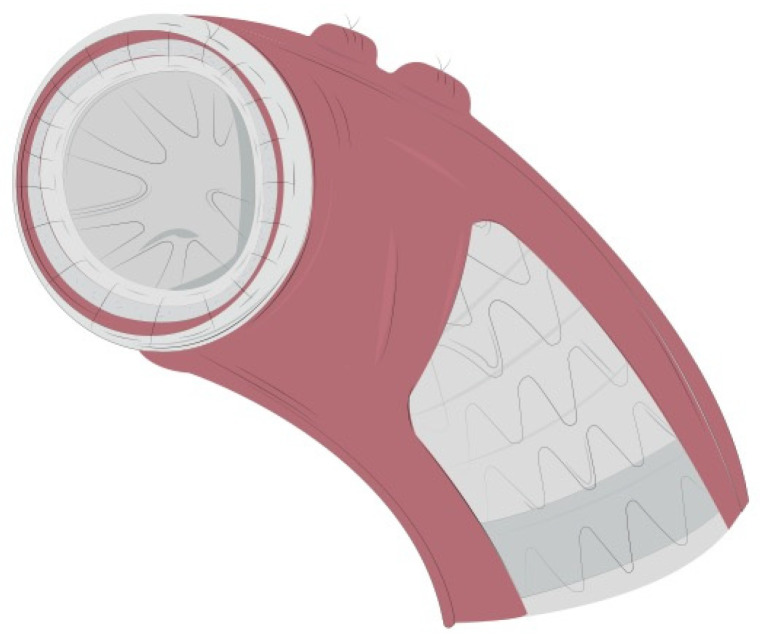
The stent graft was further fixed to the thoracic aorta in zone 1. Two large strips of Teflon were used to reinforce this suture both internally and externally. At first, a mattress 4/0 polypropylene suture was performed, followed by a running suture line that should include the Teflon felts, the stent graft and the native aorta so that all these structures are firmly joined together.

**Figure 3:**
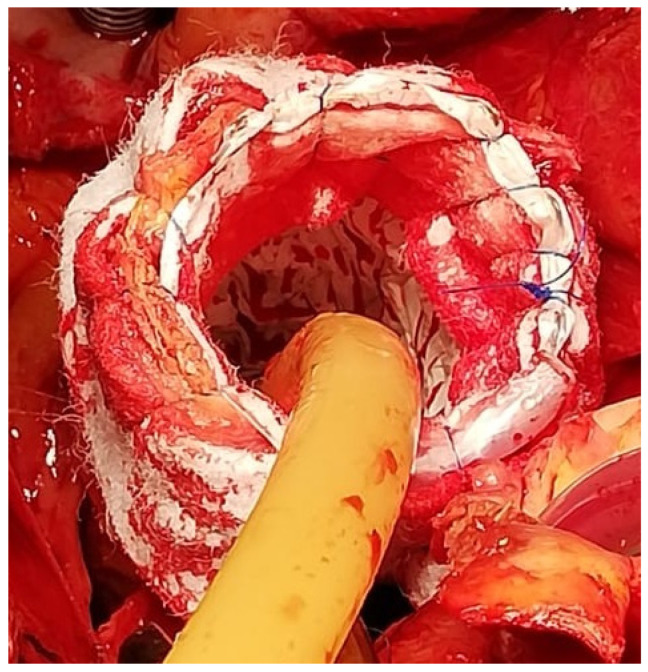
Intraoperative detail of the 4-layer distal stump. The C-TAG is well deployed into the distal aortic arch. The mattress and the running suture lines incorporating the 4 layers (Teflon-endoprosthesis-aorta-Teflon) are clearly visible.

The stent graft was further fixed to the thoracic aorta in zone 1. Two large (7–8 mm) Teflon strips were used to reinforce this suture line both internally and externally. At first, a mattress 4/0 polypropylene suture was performed, followed by a running suture line that should include the Teflon felts, the stent graft and the native aorta so that all these structures are firmly attached (Figure 2 and Figure 3).

**Figure 4:**
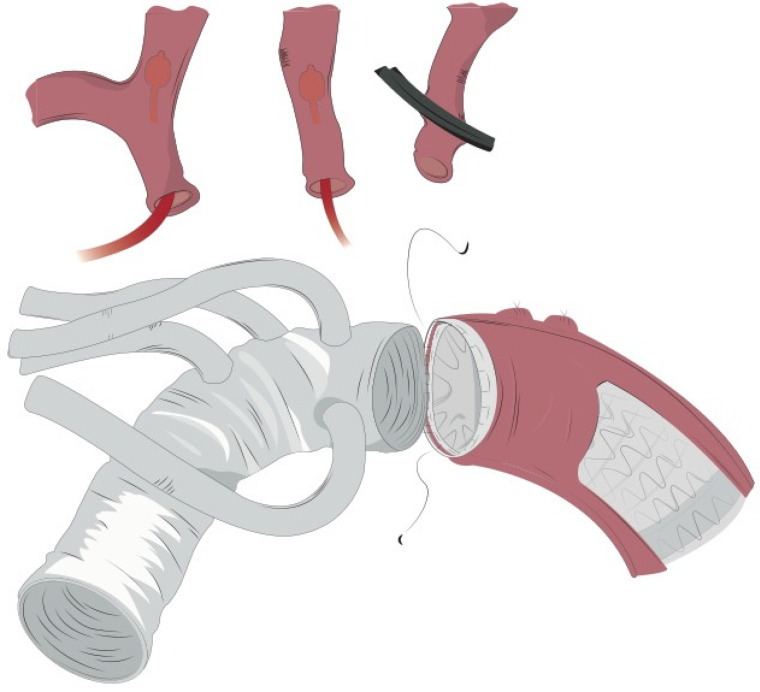
Once distal reconstruction was completed, the 4-branched graft was anastomosed to the distal stump with a 4/0 polypropylene running suture.

Once distal reconstruction was completed, the 4-branched graft was anastomosed to the distal stump with a 4/0 polypropylene running suture and cannulated through its side branch in order to start antegrade CPB (Figure 4). Secondly, the third branch of the graft was anastomosed to the left subclavian artery. In the first patient, the left subclavian was dissected and was severely fragile; therefore, the third branch was anastomosed in an end to side fashion to the axillary artery as an intrathoracic extra-anatomic bypass. 

**Figure 5:**
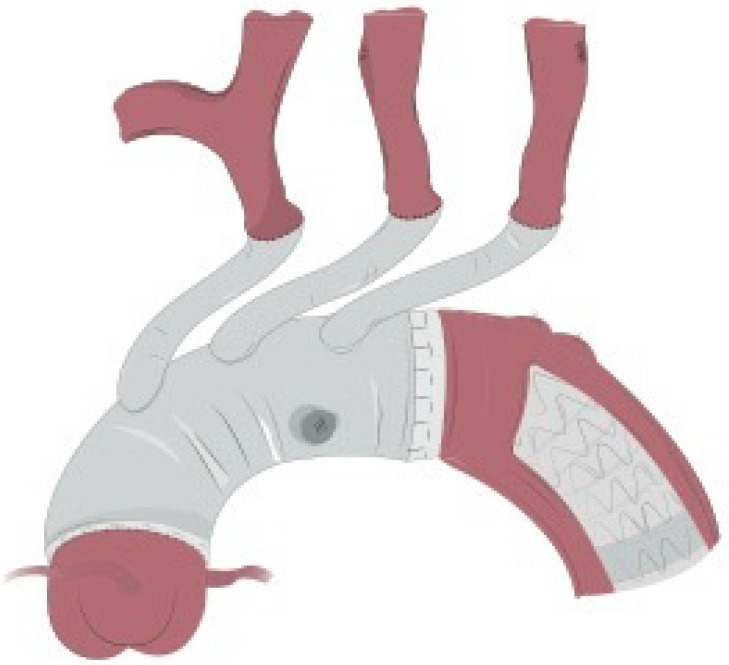
Final result of total arch reconstruction.

Rewarming was then initiated; the proximal anastomosis was then carried out. Finally, the second and first branches of the graft were anastomosed to the left carotid and to the innominate artery, respectively (Figure 5). 

Cardiopulmonary bypass times were 187 min, 232 min and 220 min, respectively. Antegrade selective cerebral perfusion (ASCP) times were 100 min, 128 min and 132 min, while hypothermic circulatory arrest times (with partial visceral perfusion) were 42 min, 54 min and 45 min. In all patients, as soon as the stent graft was deployed, a Foley catheter was inflated in the endoprosthesis and a low-flow (1–2 L per minute) perfusion through the femoral artery cannula was initiated in order to minimise the risks of visceral ischaemia. 

Intensive care unit (ICU) stay was 8, 7 and 7 days, respectively; there were no early or late deaths; moreover, no stroke or minor neurologic events occurred in the three patients. No renal failure was observed in our small cohort. 

**Figure 6:**
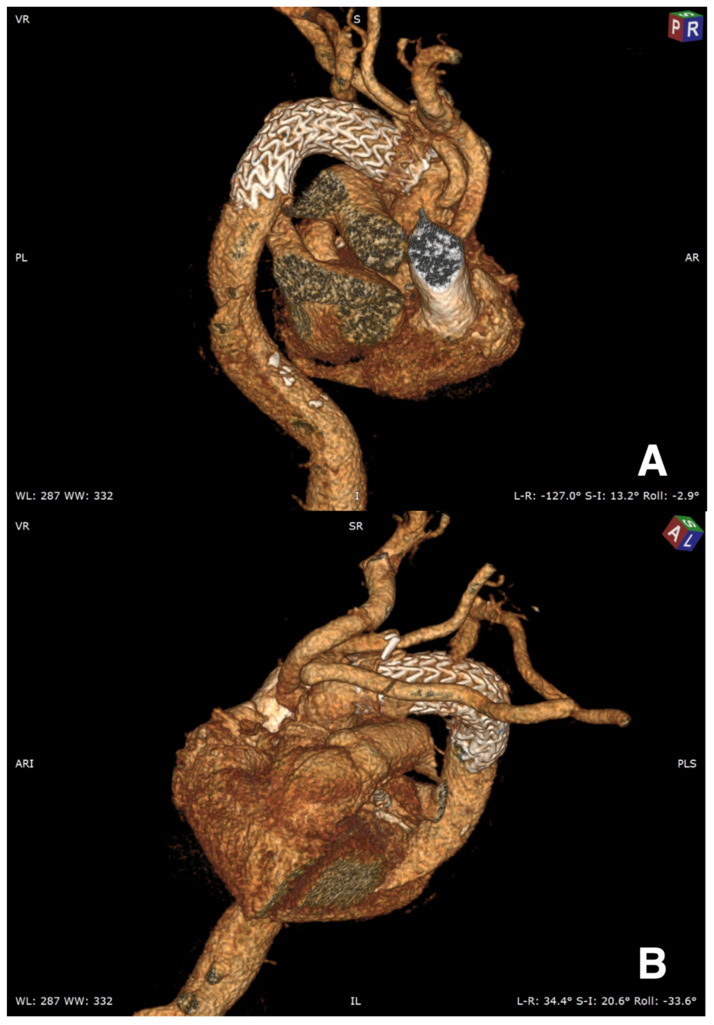
Postoperative 3D CT scan reconstruction. (**A**) Zone 1 Teflon-reinforced distal anastomosis. (**B**) Supra-aortic vessels reconstruction with left subclavian artery extra-anatomic implant.

**Figure 7:**
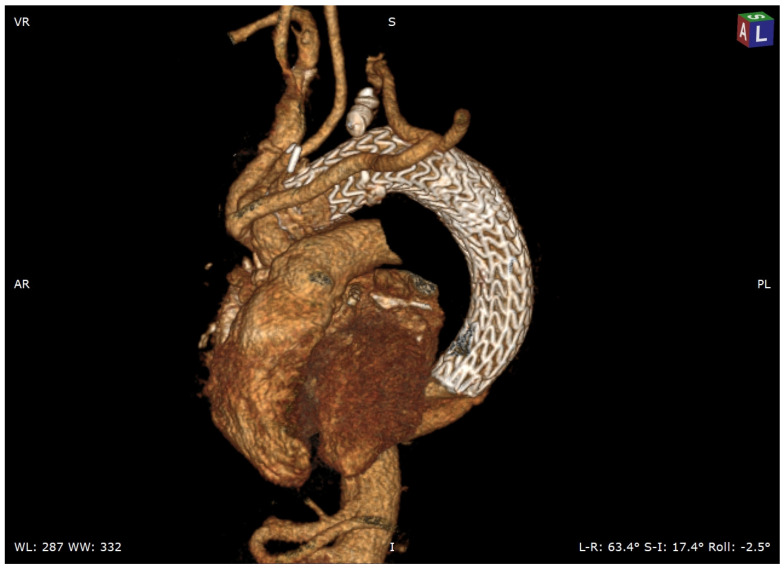
Three-dimensional CT scan: final result after endovascular distal extension and left subclavian artery origin plug occlusion.

Three months CT scan for patients 1 and 2 showed a persistent perfusion of the false lumen in the proximal descending aorta; therefore, an additional endoprosthesis was inserted (Figure 6A,B and Figure 7). Nine months follow-up confirmed total false lumen thrombosis. Three months CT scan for the third patient showed a very satisfactory result.

### 2.5. Total Endovascular Frozen Elephant Trunk

Today, with ever increasing technical innovations, the possibility of approaching aortic arch pathologies via a total endovascular route has become feasible. The combination of multiple endovascular devices addressing the aortic arch with its branches allows for a complete exclusion of aneurysmatic/dissected portions of the aorta, keeping native epiaortic vessel anatomy and blood flow [21]. 

## 3. Discussion

The main goal of emergency surgery for acute type A aortic dissections is to prevent aortic wall rupture. Therefore, many surgeons advocate exclusively performing the essential ascending aortic replacement, with or without replacement of the proximal arch. Shresta and colleagues as well as Kazui and colleagues [19,20,21,22] recommended a more aggressive strategy, with total aortic arch replacement, in order to improve late surgical outcomes. Such an aggressive initial approach may be especially advisable in a setting where there is high suspicion (or even more so in case of confirmed diagnosis) of a collagen disorder such as Marfan syndrome [23]. However, total aortic arch replacement in acute aortic dissection is without a doubt a technical challenge.

The essential steps of our procedure include implantation of a Gore C-TAG endovascular prosthesis into the proximal descending aorta, Teflon-reinforced suture of the graft to the native aortic wall (usually in zone 1), total arch replacement with a 4-branched vascular graft, antegrade selective cerebral perfusion and moderate hypothermic circulatory arrest. 

Spinal cord ischaemia (SCI) is a known drawback of surgery involving the aortic arch and descending aorta. Although the rate of SCI is low (approximately 5%) [6], the potential consequences in the early and late postoperative period may severely impact the patients’ quality of life.

In our institutions, on the basis of the experimental work by Biglioli et al. [24], we believe that, in all cases, selective reimplantation of the left subclavian artery plays a significant role in preventing late SCI, especially in emergent cases in which cerebral vascular anatomy may not be known. Moreover, as recently highlighted by Di Eusanio et al. [25], we can perform antegrade or retrograde aortic low-flow perfusion during systemic circulatory arrest. In this way, we aim to prevent, accompanied by moderate hypothermia, visceral hypoperfusion and lower SCI.

For the time being, there are no single hybrid prostheses for the FET procedure that are commercially available worldwide, while a wide selection of off-the-shelf Gore endovascular grafts and vascular prostheses are available in the majority of cardiothoracic operating units.

Our technique relies on the same endovascular skills necessary for the implantation of any FET hybrid prosthesis, therefore avoiding the learning curve for “pure” endovascular technology, and the use of fluoroscopy at the time of the operation that would require a hybrid operating theatre.

In conclusion, we describe a technique that does not require a ready-made hybrid prosthesis; therefore, we can have an individually tailored approach for each patient, combining any possible stent graft with the appropriate vascular graft, leaving the surgeon free to use his/her preferred total arch configuration and position of the distal anastomosis.

Dr. Coselli underlined the great versatility of the Y-graft to accommodate for arch anomalies or to modify the sequence of anastomoses according to intraoperative findings. The trifurcated graft may be tailored to the exact configuration of the patient’s anatomy, thus reducing the potential for kinking that may be an issue with the 4-branched graft technique, in which the position of the branches is fixed [26].

Although our small series of patients consists of acute aortic dissections only, this technique could be applied to any other aortic arch pathology, such as chronic dissections or aneurysms. Moreover, we believe that, because of the individually tailored approach and widespread availability of the necessary materials, this technique can reveal itself useful in many different operative scenarios. 

## 4. Conclusions

We described our frozen elephant trunk technique alongside the broad spectrum of surgical possibilities for approaching aortic arch pathologies. Moreover, our results so far show comparable intraoperative times and rate of postoperative comorbidities when compared to other techniques. Even though our case series only includes acute aortic dissections, we believe our tailored approach could be applied to any acute or chronic aortic arch pathology. 

In addition, a small cohort of patients and relatively short follow-up times warrant further investigation in terms of postoperative survival, future aortic events and remodelling of the residual aortic dissection.

## Data Availability

Data available on request due to privacy and ethical restrictions. The data presented in this study are available on request from the corresponding author. The data are not publicly available because of they are part of the patients’ medical and surgical records.
